# Towards a Physarum learning chip

**DOI:** 10.1038/srep19948

**Published:** 2016-02-03

**Authors:** James G. H. Whiting, Jeff Jones, Larry Bull, Michael Levin, Andrew Adamatzky

**Affiliations:** 1 International Centre in Unconventional Computing, University of the West of England, Bristol, UK; 2 Tufts Center for Regenerative and Developmental Biology, Tufts University, Medford, USA

## Abstract

Networks of protoplasmic tubes of organism *Physarum polycehpalum* are macro-scale structures which optimally span multiple food sources to avoid repellents yet maximize coverage of attractants. When data are presented by configurations of attractants and behaviour of the slime mould is tuned by a range of repellents, the organism preforms computation. It maps given data configuration into a protoplasmic network. To discover physical means of programming the slime mould computers we explore conductivity of the protoplasmic tubes; proposing that the network connectivity of protoplasmic tubes shows pathway-dependent plasticity. To demonstrate this we encourage the slime mould to span a grid of electrodes and apply AC stimuli to the network. Learning and weighted connections within a grid of electrodes is produced using negative and positive voltage stimulation of the network at desired nodes; low frequency (10 Hz) sinusoidal (0.5 V peak-to-peak) voltage increases connectivity between stimulated electrodes while decreasing connectivity elsewhere, high frequency (1000 Hz) sinusoidal (2.5 V peak-to-peak) voltage stimulation decreases network connectivity between stimulated electrodes. We corroborate in a particle model. This phenomenon may be used for computation in the same way that neural networks process information and has the potential to shed light on the dynamics of learning and information processing in non-neural metazoan somatic cell networks.

Most work in biology and bio-inspired engineering focuses on neural systems as the medium for memory and computation in living tissues. However, neural-like information-processing, decision-making, and learning have been reported in a wide range of systems well beyond the traditional CNS, including sperm^1^, amoebae^2^, yeast^3^, and plants^4^,^5^,^6^,^7^,^8^,^9^,^10^. These appear to be mediated by well-conserved, ubiquitously-present mechanisms that appear to be also involved in neural information processing, such as the cytoskeleton^11^ and electrical signaling^12^,^13^. Single somatic cells perform subtraction, addition, low- and band-pass filtering, normalization, gain control, saturation, amplification, multiplication, and thresholding^14^. It is becoming clear that neural networks have no monopoly on such functions, and indeed fascinating examples of memory and neural-like dynamics have been found in the immune system^15^,^16^, bone^17^,^18^, heart^19^,^20^, and physiological disorders such as diabetes^21^. A better understanding of fundamental aspects of computation and memory in living tissues (not limited to the special case of nerve networks) would have implications not only for evolutionary biology, but also for regenerative biomedicine, synthetic bioengineering, and the information sciences^22^. Thus, it is important to study tractable non-neural systems in which plasticity and learning can be mechanistically dissected. One such versatile model is represented by the slime moulds.

The plasmodial stage of the slime mould *Physarum polycephalum* is a macroscopic multinucleate single celled organism, it is visible by the naked eye as a yellow mass and or network of strands which may span tens of centimetres^23^. The strands are protoplasmic tubes which are extended to forage for food matter; these tubes move by a process known as shuttle streaming, where cytoplasm is forced rhythmically back and forth towards a desirable location^24^. The frequency of this streaming is determined by the environmental conditions such as temperature and degree of attraction to a food source; as such it may change as new stimuli are sensed^25^.

Computing with biological substrates is a relatively young topic of research, however much progress has been made in recent years. The slime mould *Physarum polycephalum* has been the centre of attention for a number of years as it has been suggested that the organism can be used to compute various complex tasks^26^,^27^. The organism follows fairly basic behavioural patterns, growing towards food and away from light^28^. This innate behaviour can be interpreted as computation when inputs and outputs of a function are given as data in the form of spatial configurations of food and other environmental conditions that can be understood by the organism^27^; using this analogy, various computational operations have been demonstrated. *Physarum polycephalum* can solve shortest path and other network problems^29^,^30^,^31^, solve mazes and has been shown to closely approximate human transport networks on flat^32^ and 3D terrain^33^. It has been suggested that the apparent intelligence of the slime moulds is an observed phenomenon called emergent intelligence^34^; seemingly complex behavior emerges from a set of biochemical rules whose actions are the result of a set of interpretations of their environment made at the cellular level. This is why when applying multiple positive stimuli, the organism appears to make decisions on which stimuli to respond to; sensitive receptors in the cell detect various changes in the environment and act accordingly. While the organism is not inherently intelligent, we can take advantage of its environmental response and apply criteria of computation which approximate responses of traditional computation such as those aforementioned shortest path approximations which merely take advantage of the foraging behavior of the organism.

Logic gates have also been produced using *P. polycephalum*:

- First recognisable Boolean logic gate using propagation of plasmodial *P. polycephalum* along agar channels whose geometry was encoding the Boolean functions^34^.
- Ballistic based approach to implement logic gates on agar geometry and simulated a one-bit half-adder using combinations of individual gates into one geometry^27^.
- Experimentally derived model of plasmodial propagation on agar and simulated a 1 bit half adder^35^.
- Logic gates based on microfluidic properties of protoplasmic tubes of the slime mould, meaning agar geometry was no longer a constraint^36^.
- Logic gates using environmental stimuli as inputs and measured the slime mould’s bioelectric response^28^,^37^, a process which took minutes rather than hours to compute a logical operation.
- Logic gates with light inhibition as a repellent^38^.
- Quantative transformation of inputs for simplification of computation in unconventional computing substrates such as *P. polycehpalum*^39^.

The recent surge of interest in this topic has also led to other discoveries; there have been a number of publications regarding the electrical interface of protoplasmic tubes with electronic components for the purpose of novel biosensors and bioelectronics. Biosensors have been produced which detect chemicals in the environment^40^,^41^, tactile stimulation^42^,^43^ and colours of light^44^. These sensors have the benefit of a very long shelf life, and can grow on a variety of substrates; advantages over bacterial and fungal biosensors have been previously discussed in detail^40^. The slime mould has interesting properties, it is conductive with apparent low-pass filter properties^45^,^46^ and can be used as an electronic component. The protoplasm has also been interfaced with an FPGA to facilitate implementation of arithmetic calculations and bioelectric measurement^47^. It has been shown that adding nano-particles can alter the conductive properties of protoplasmic tubes^48^,^49^; this concept has been extended into producing working electronic components with funtionalised protoplasmic tubes of *P. polycephalum*^50^.

It is the purpose of this paper to report findings of a programmable grid of protoplasmic tubes which can change morphology in response to electrical stimulation.

## Method

Plasmodium of *P. polycephalum* was cultured from dried sclerodia on filter paper; re-hydrating the sclerotia and placing it on 2% non-nutrient agar in a 9 cm diameter Petri-dish (Fisher Scientific, UK). The plasmodium was fed daily with several rolled oat flakes; the culture was transplanted to a fresh agar Petri-dish weekly to minimise unwanted microbial growth.

**Figure 1:**
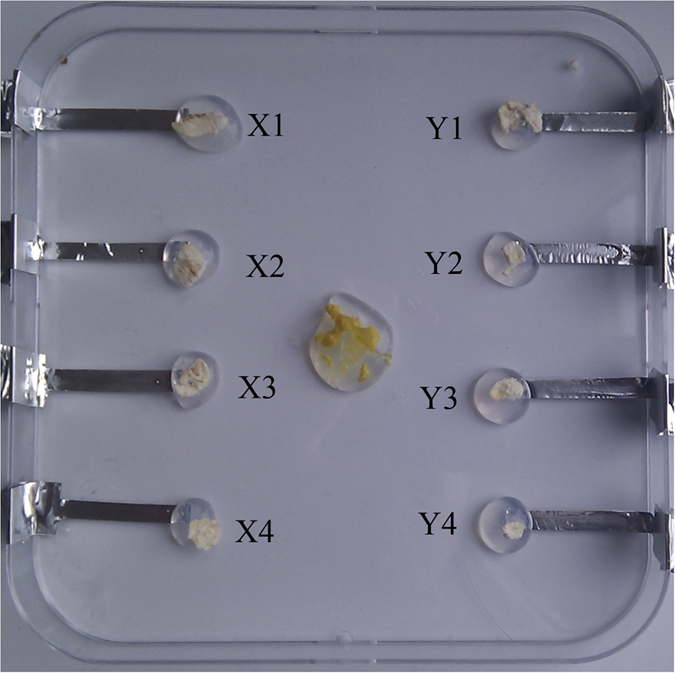
The unconnected grid array, seeded with plasmodium on the center agar hemisphere, from which it grows out and connects each tube.

The programmable grid of plasmodial connections is produced using agar as a supporting medium, aluminium tape electrodes and a 12 cm square Petri-dish; this set up is shown in Fig. 1. From the 10 mm wide aluminium tape (RS Components, UK), thin 5mm sections are cut which are placed in the Petri-dish, 4 electrodes are placed on the left of the Petri-dish and 4 on the right, these are spaced 3 cm apart and 6 cm across from each other. On the tip of each tape electrode, 1 ml of 2% non-nutrient agar is placed to maintain the organism at each electrode; 2 ml of agar is placed in the centre of the Petri-dish to help facilitate protoplasmic tubes to span the gaps between every electrode. A rolled oat flake is placed on top each agar electrode to provide a source of nutrients and to act as an attractant to induce the protoplasmic tubes to reach every electrode.

**Figure 2:**
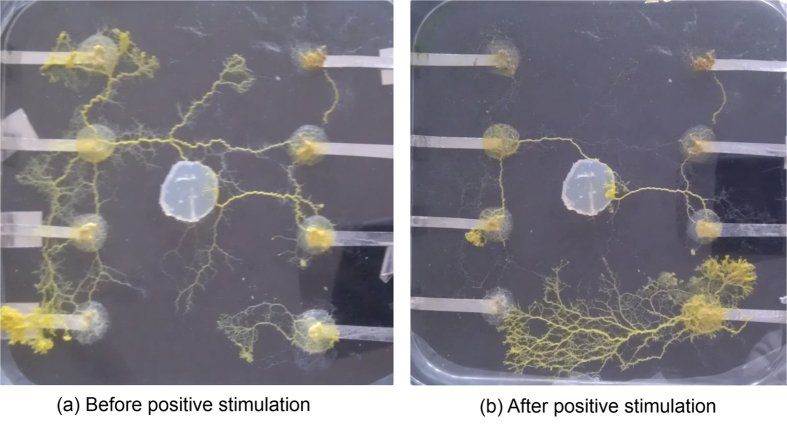
The programmable grid array connected by protoplasmic tubes of *Physarum polycephalum* clearly visible as yellow tubes connecting the agar electrodes. High network connectivity is visible before LALF stimulation between X3 and Y3; after stimulation the network connectivity remains between the stimulated electrodes while other connections have been abandoned.

Plasmodium taken from the culture is placed on the centre agar, from which it may grow and colonise every electrode; fully grown connections are demonstrated in Fig. 2. To facilitate optimum growth conditions, the lid of the Petri-dish was placed over a source of steam until it was saturated with condensate water; the lid is then placed on the base and sealed with Para-film to maintain humidity and sterility. The Petri-dish should then be placed in a dark location of approximately 20 degrees Celsius and allowed to grow until it has colonised every agar hemisphere; this process should take between 2 and 4 days.

In order to mimic a learning system, one must apply teaching rules to change the state of the system; in order to stimulate or inhibit the organism, we must stimulate the grown network of protoplasmic tubes. It is known that heat, food sources and light all induce positive and negative stimulation effects, however in a closed loop system they are more complex and difficult to control. It was hypothesised that a sinusoidal voltage may also be used as a stimulant for the protoplasmic tubes, and is tested in this paper. High amplitude high frequency (HAHF) voltage stimulation between two electrodes causes the tube to be abandoned while low amplitude low frequency (LALF) voltage stimulation causes increased growth between electrodes and abandonment of non-stimulated electrodes; HAHF voltage was 2.5 V peak-to-peak at 1000 Hertz, while LALF voltage was 0.5 V peak-to-peak at 10 Hertz. Both voltages were produced using an Aim-TTi TG550 Function Generator (RS Components, UK). In order to test this stimulation, 10 HAHF and 10 LALF experiments were run using fully grown Petri-dishes of protoplasmic tubes; the voltage was applied between a left and right electrode, both chosen at random for each experiment. To determine if stimulation had any effect on the tube, the resistance of each left to right tube network was measured before and after stimulation to detect changes in tube thickness and connectivity; the stimulation was applied for a total of 12 hours.

The result of stimulation is a function of the resistance (*R*), before and the stimulation type between given electrodes. On the left we have *X*_1_…, *X*_4_ and on the right we have *Y*_1_…, *Y*_4_, so 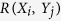, 1 ≤ *i*, *j* ≤ 4. Measuring each combination allows us to determine if the resistance between the stimulated electrodes is significantly different to those not stimulated, for both types of stimulation.

## Results

**Figure 3:**
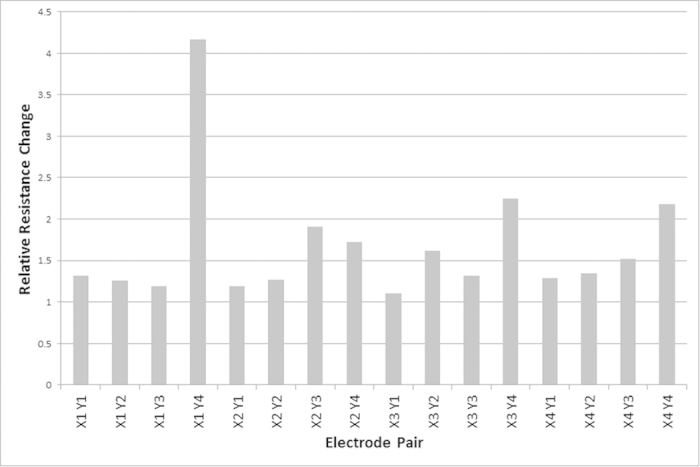
An example of high amplitude high frequency voltage stimulation after 12 hours. Stimulation occurred between electrodes X1 and Y4.

In 10 repetitions of HAHF stimulation, 8 experiments showed a marked increase in resistance in the network between the stimulated electrodes while the resistance between non-stimulated electrodes was similar to the starting resistance. In the remaining 2 experiments, the resistance between the stimulated electrodes was comparable to the unstimulated electrodes. Statistical analysis was performed using the two-tailed, Mann-Whitney U test; a 95% confidence interval was used, with statistical significance demonstrated by a p value of 0.02. An example output is demonstrated in Fig. 3 where the relative resistance is calculated from the start and finish resistances; stimulation occurred between electrodes *X*_1_ and *Y*_4_ and it can be seen that the relative resistance change is twice as high at this electrode pair compared to the other electrode pairs.

**Figure 4:**
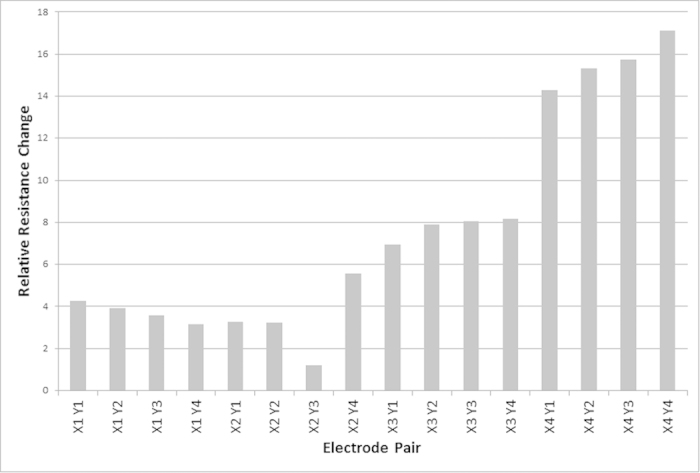
An example of low amplitude low frequency voltage stimulation after 12 hours. Stimulation occurred between electrodes *X*_2_ and *Y*_3_.

In 10 repetitions of LALF stimulation, 7 experiments showed a marked increase in resistance for the electrodes that were unstimulated; the resistance between stimulated electrodes remained similar to the starting resistance. In 1 experiment, the resistance between all electrodes remained relatively similar to the starting resistances; in the remaining 2 experiments, all resistances increased. Statistical analysis was performed using the two-tailed, Mann-Whitney U test; a 95% confidence interval was used, with statistical significance demonstrated by a p value of 0.042. An example output is demonstrated in Fig. 4 where the relative resistance is calculated from the start and finish resistances; stimulation occurred between electrodes *X*_2_ and *Y*_3_ and it can be seen that the relative resistance change is between 3 and 16 times lower at this electrode pair compared to the other electrode pairs.

**Figure 5:**
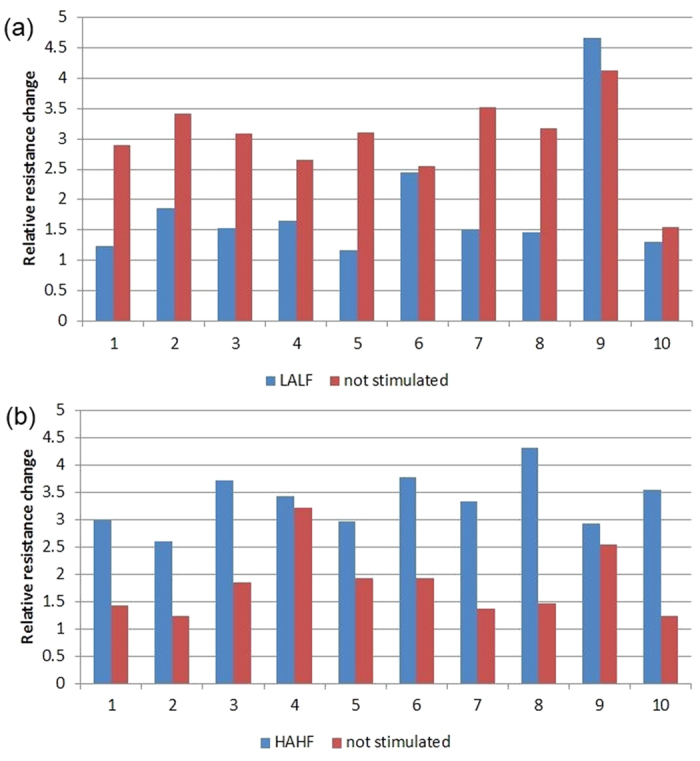
Relative changes in resistance with LALF (a) and HALF (b) stimulation. Each pair of columns represents the relative resistance change for one experiment.

The paired output for 10 repetitions of LALF (a) and HALF (b) stimulation are shown in Fig. 5, demonstrating the difference in relative change in resistance of the stimulated tube and the mean relative change of the unstimulated tubes.

### Modelling Experiments

The experimental results presented in this paper show promising steps towards the goal of controlling the shape of the body plan of *Physarum* plasmodium at will. However, because it is a living organism, the plasmodium is relatively unpredictable and difficult to directly control. We must turn to modelling to explore the potential of dynamically ‘re-wiring’ the organism. We can frame the LALF and HAHF stimuli as generic +ve and −ve stimuli respectively. These stimuli affect the behaviour of the plasmodium directly at the spatial locations where the stimuli are presented. These local stimuli then affect the *global* pattern of the plasmodial network as the organism adapts its body plan in response to the stimuli.

We use the multi-agent model of *Physarum* introduced in^51^. The purpose of the model is to demonstrate how the complex behaviours of *Physarum* can emerge from very simple components and their local interactions–using no specialised component parts. The model has been successful in reproducing the biological behaviour of the *Physarum* plasmodium, including its dynamical pattern formation phenomena^52^, oscillatory phenomena^53^,^54^, coarsening phenomena^55^ and response to electrical stimuli^56^. From a computational perspective the model can reproduce the spatially represented embedded unconventional computations approximated by the organism (for more information, see^57^).

This modelling approach uses a population of indirectly coupled mobile particles with very simple behaviours, residing within a 2D diffusive lattice which stores particle positions and the concentration of a generic diffusive factor referred to as chemo-attractant. Particles deposit this chemo-attractant as they move and also sense the local concentration of chemo-attractant, orienting their position to face the strongest source. The particle population spontaneously forms self-organised networks corresponding to Turing-type Reaction-Diffusion patterns^58^. These networks also exhibit quasi-material behaviours such as spontaneous branching of new networks or network minimisation. The type of network (for example, highly reticulated, or strongly minimising) can be selected by altering two main sensory parameters^58^. These parameters are the particle sensor angle (*SA*) and particle rotation angle (*RA*). These parameters affect the area coupling the particles and also the response of the particles to local concentration changes. A third parameter (*SO*) specifies the distance of the particles’ sensors from its location in space and acts as a scaling parameter for the resultant networks. Collective particle positions represent the global pattern of the plasmodium and collective particle motion represents flux within the plasmodium. The particles act independently and iteration of the particle population is performed randomly to avoid any artifacts from sequential ordering.

The evolution of the model transport network can be influenced and constrained by externally presented spatial stimuli. The stimuli are projected onto the 2D lattice as real valued numbers. The stimuli from concentration gradients within the lattice. Diffusion within the lattice is implemented by a simple mean filter kernel (in this case, size 5 × 5). The mean value is multiplied by a damping factor (0.9) to limit the diffusion distance of the chemo-attractant within the lattice. Positively weighted stimuli correspond to chemo-attractants and act to attract nearby particles and also constrain the evolution of the particle networks. Negatively weighted stimuli correspond to chemo-repellents and repel nearby particles, causing the network to avoid these locations.

There is no explicit representation of electrical stimulation of the plasmodium in the model. However in^56^ we found that we could reproduce the short-term and long-term changes in electrical potential within the plasmodium by using the generic attractant stimuli to alter flux within the particle networks. To reproduce the effect of the LALF (+ve) and HAHF (−ve) stimuli we can use differences in attractant concentration to alter network behaviour.

Because the plasmodial networks in the experimental array with 8 stimulus locations were relatively simple in their structure (2), for the modelling experiments we used a slightly more complex array consisting of 25 stimulus points in a 5 × 5 regular pattern. This allows the effect of changes in spatial stimuli to be more easily observed.

**Figure 6:**
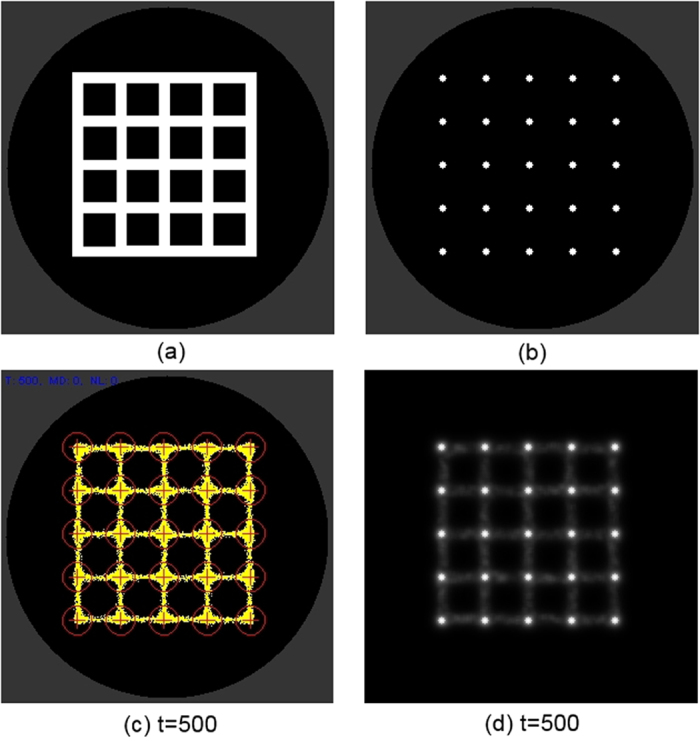
Setup of modelling experiments. (**a**) virtual Petri dish (grey) and grid mask (white) to initialise particles, (**b**) location of 5 × 5 stimulus locations, (**c**) stabilised particle positions with sensor regions indicated by circles, (**d**) greyscale indication of flux within the particle population and attractant sources (lighter points).

We used a fixed size population consisting of 8000 particles to represent the *Physarum* plasmodium. Particle parameters were *SA* 60, *RA* 60 and *SO* 9 and the deposition level for all particles was 5 units per step. The population was inoculated and confined in a 5 × 5 grid mask pattern (Fig. 6a) by means of strong attractant stimuli. The grid pattern was then removed, leaving only a the 5 × 5 point source array stimuli (Fig. 6b). The default level of stimuli at the array nodes was 5 units per scheduler step, matching the trail value deposited by the particles. The structure of the model plasmodium is shown in Fig. 6c which also indicates the regions in the array (circled) used to measure the particle population size at each node point in the array. This region was a 13 × 13 square window centred around each stimulus point. The spatial pattern flux within the particle population in the diffusive lattice is indicated in Fig. 6d where the grey lines are particle flux and the bright point sources represent stimulus locations.

### Model Response to Addition and Removal of +ve Stimuli

**Figure 7:**
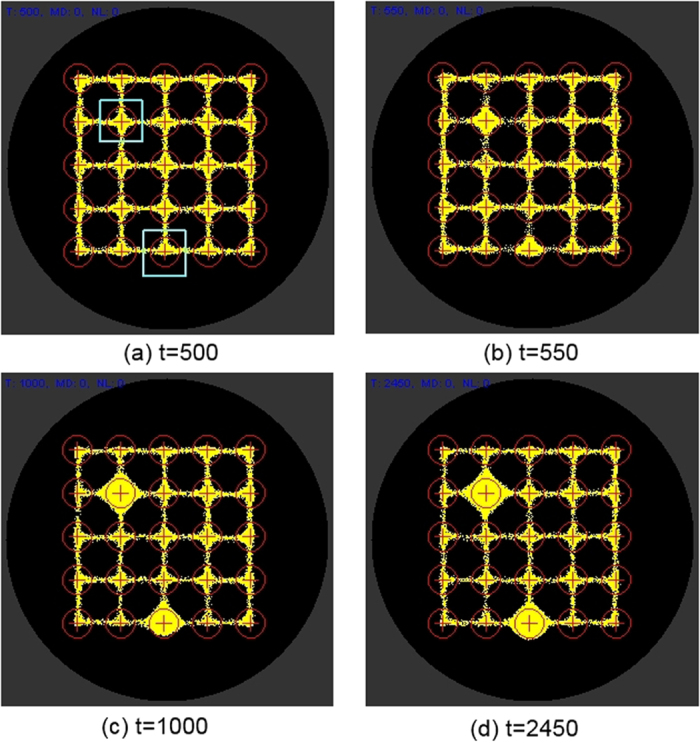
Effect of +ve stimuli on particle network distribution. (**a**) +ve stimuli introduced after t = 500 at nodes column, row (2,2) and (3,5) (pale square regions), (**b**–**d**) particles from nearby areas are attracted to these stimulated nodes.

Figure 7 indicates the response of the particle model to +ve stimuli at two nodes located at positions (2,2) and (3,5) where *(c,r)* indicates column number and row number respectively (nodes annotated with pale square border). When these nodes are presented with +ve stimuli (500 units per scheduler step, compared to 5 units for particle movement deposition) particles are drawn to these nodes from nearby nodes. When lower values are used for the +ve stimuli sources, the effect is migration to these nodes is the same but less pronounced (not shown).

**Figure 8:**
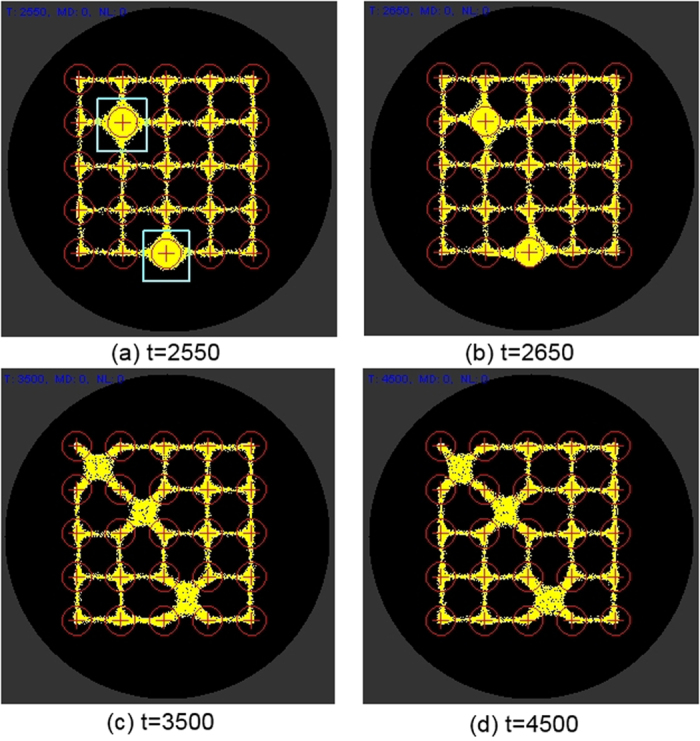
Removal of +ve stimuli redistributes particle distribution to nearby nodes. (**a**) +ve stimuli removed after t = 2500 at nodes column, row (2,2) and (3,5) (pale square regions), (**b–d**) particles from previously stimulated nodes are redistributed between adjoining nodes.

**Figure 9:**
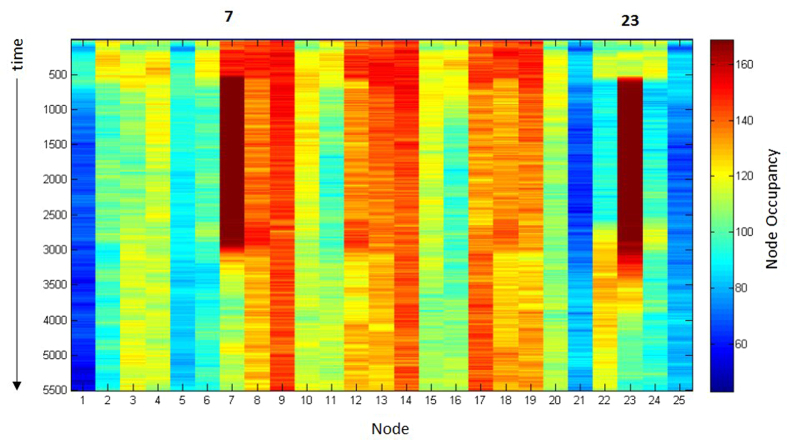
Space-time plot of particle population flux at 25 nodes of 5 × 5 array. Time proceeds downwards, x-axis is array node number, y-axis is time. Nodes 7 and 23 received +ve stimuli at 500 steps which was removed after 2500 steps. Greater ‘warmth’ (online) indicates increase in flux.

The removal of +ve stimuli from (2,2) and (3,5), which returns them to the same bacground level as the remainder of the array, causes an efflux of particles away from these nodes (Fig. 8). The particles are redistributed amongst nearby nodes (Fig. 8d). The flux of the particle population during the addition and removal of +ve stimuli can be observed in Fig. 9, which shows a space-time plot of changing population size at each node point. The stimulated nodes correspond to columns 7 and 23 in the chart and the ‘temperature’ based changes in brightness (online) correspond to increases (hotter) and decreases (colder) in flux respectively. The chart shows that when +ve stimuli are initiated at nodes 7 and 23, the population at these nodes increases, attracting particles from nearby nodes. When the stimuli are removed, the flux through these nodes decreases again whilst the flux near neighbouring nodes increases.

**Figure 10:**
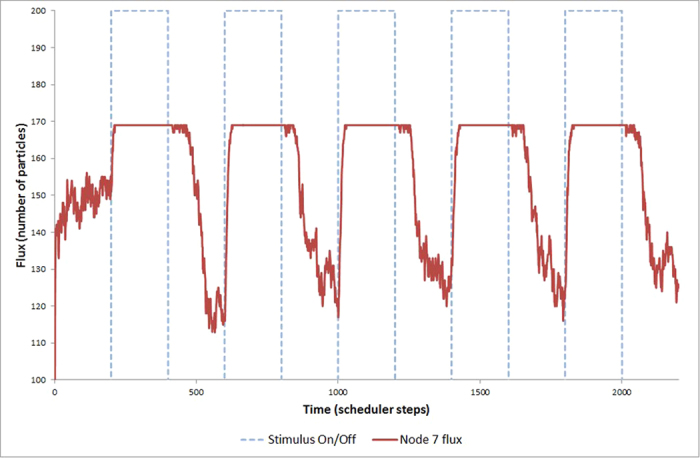
Repeated stimulus at same location results in increase in baseline occupancy. Plot shows regular periodic stimulation (dashed line) at node 7 of 25 (as in Fig. 7). Solid line shows plot of node 7 occupancy at periods of stimulation and baseline occupancy. Note how baseline non-stimulated activity rises over repeated applications.

Repeated application of +ve stimuli at the same location causes a gradual redistribution of particles to nodes nearby the stimulus point. This redistribution ensures that the baseline ‘activity’ (occupancy) around the node increases even when the node is not being stimulated. A plot of the increased occupancy after repeated stimulus for node 7 is shown in Fig. 10. The stimulus on and off periods are indicated by the dashed lines (blue, online) and the baseline activity (measure of particle flux at the node) is represented by the solid line (red, online). This increase in baseline activity after repeated stimulation may be interpreted as a primitive spatially implemented memory of the stimulus.

### Model Response to Addition and Removal of −ve Stimuli

**Figure 11:**
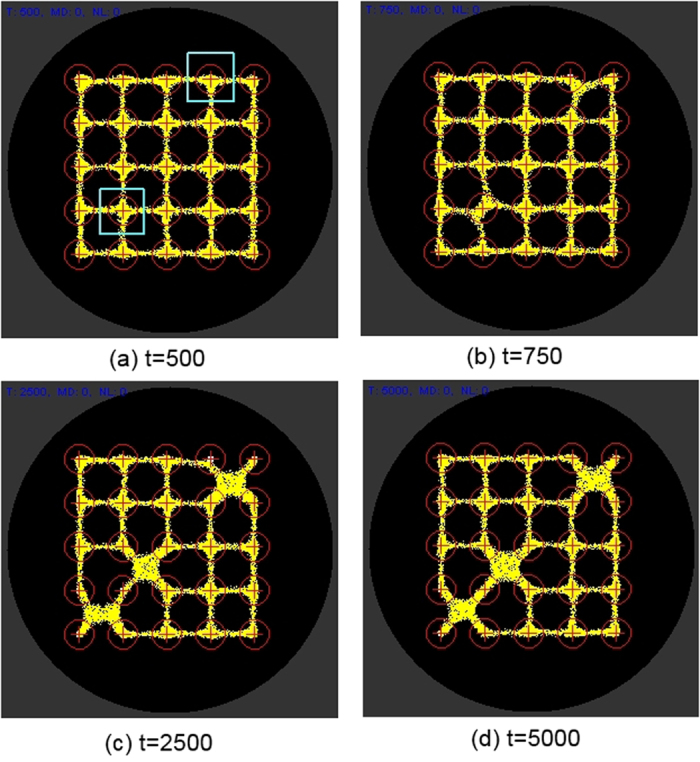
Representing −ve stimuli by lowered stimulus value at target nodes. (**a**) −ve relative stimuli introduced at column, row (1,4) and (2,4) (pale square regions), (**b–c**) particles migrate from lower valued nodes and are redistributed between adjoining nodes, (**d**) after resetting stimulus nodes to value of other nodes there is a slow recovery in occupancy.

**Figure 12:**
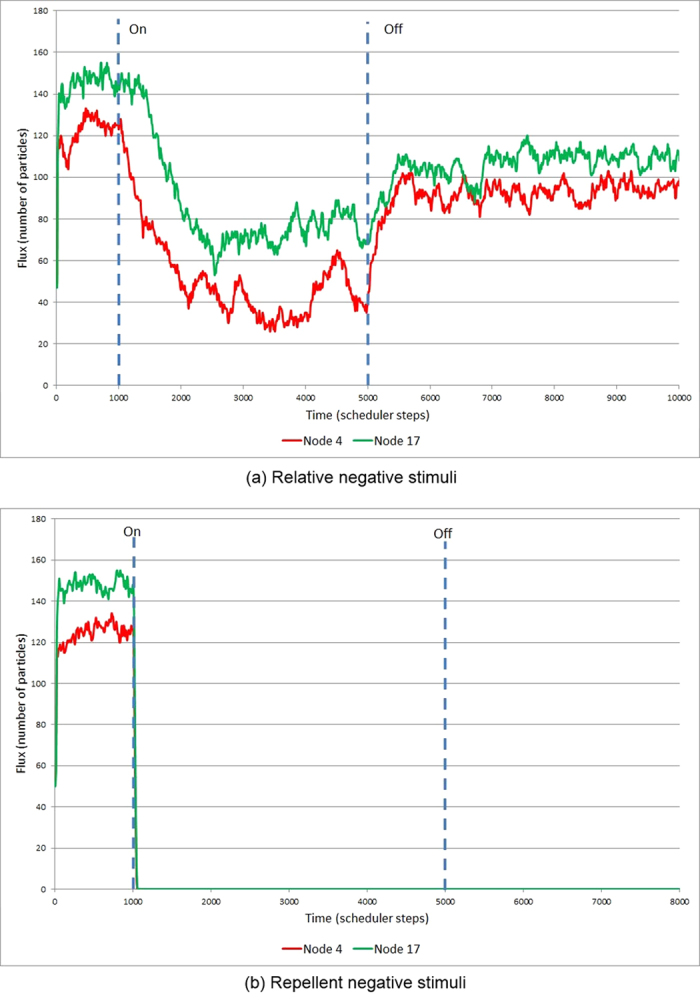
Plots of node 4 (red, online) and node 17 (green, online) flux levels in response to −ve stimuli. (**a**) plot in response to relative lower stimuli levels shows gradual fall in occupancy after activation (leftmost vertical bar). When the nodes are reset to the baseline value (rightmost vertical bar) there is a gradual increase in occupancy (see Fig. 11), (**b**) plot in response to negatively weighted repellent stimuli shows a sudden fall in occupancy after activation as particles abandon the locations of repellents. When the nodes are set to the baseline value there is no re-occupancy of the nodes (see Fig. 13) and baseline activity remains at zero.

The representation of −ve stimuli at certain nodes can be achieved in two ways. Firstly, by decreasing the projection of attractant at stimulated nodes below the baseline level of the remaining nodes (baseline stimulus level 5 units per step and target node stimulus of 1 unit per step). In this case all nodes still receive +ve stimuli but the target nodes are −ve relative to the remaining nodes. Under this condition there is a gradual fall in occupancy at the target nodes after stimulation (Fig. 11a–c). When the target nodes are restored to the same baseline value as the remaining nodes, there is a small gradual increase in occupancy (Fig. 11d). This is reflected in the plot of occupancy shown in Fig. 12a.

**Figure 13:**
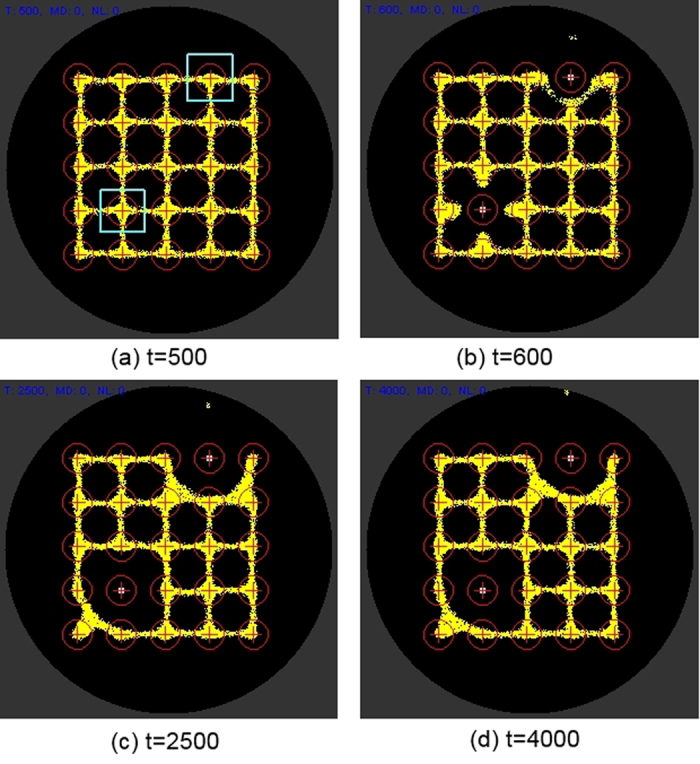
Representing −ve stimuli by negatively weighted repellent stimulus value at target nodes. (**a**) −ve negatively weighted stimuli introduced at column, row (1,4) and (2,4) (pale square regions), (**b**–**c**) particles are repelled from stimulus nodes and are redistributed between adjoining nodes, (**d**) at strong −ve stimulus levels there is no re-occupancy of nodes after resetting the target nodes to baseline values.

Negative stimuli can also be achieved by projecting (repellent) negatively weighted values at the target nodes. The response of the model plasmodium in this instance is a much quicker response as individual particles migrate away from the diffusing repellent field (Fig. 13a–c). When the −ve stimulus is at high concentrations (500 units per scheduler step) there is no re-occupancy of the target nodes even after the −ve stimulus is removed (Fig. 13d, and plot in Fig. 12b).

**Figure 14:**
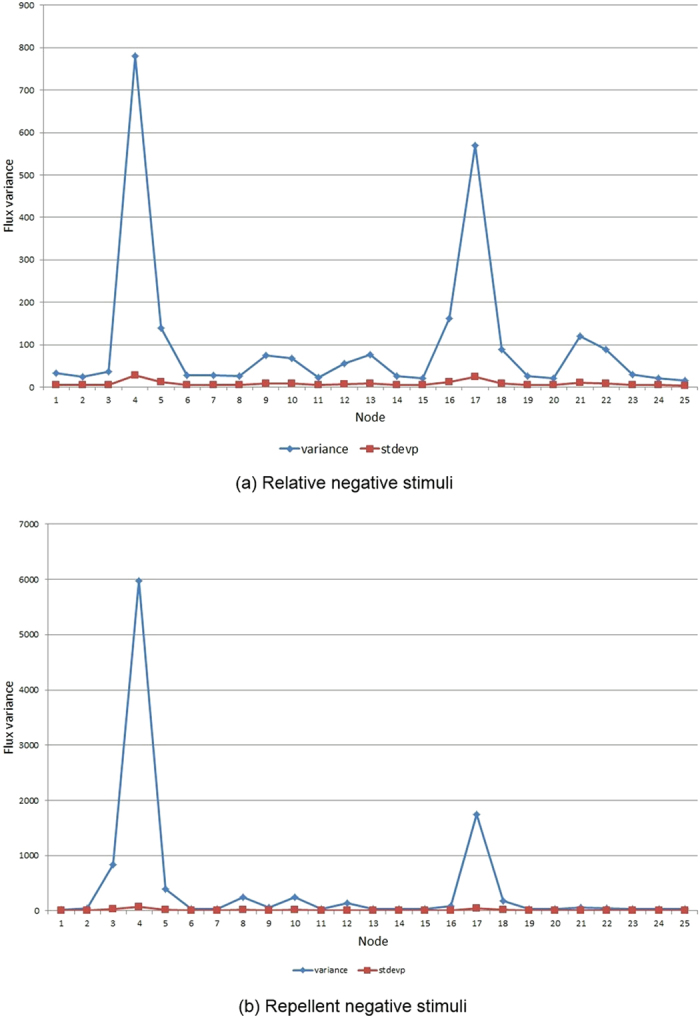
Plots of signal variance (blue, online) and standard deviation (red, online) for all nodes. (**a**) plot in response to relative lower stimuli levels shows large variance at nodes 4 and 17 compared to remaining nodes. Smaller peaks occur at nodes close to the stimulated nodes, (**b**) plot in response to negatively weighted repellent stimuli shows large variance in nodes 4 and 17 in response to repellent stimuli at these nodes. Variance at unstimulated nodes is much smaller, indicating that the stimuli are responsible for changes in flux at nodes 4 and 17.

The 23 nodes with unchanging stimulus levels are not plotted in Fig. 12 and have relatively uniform population flux at the nodes. The role of stimulus differences in changing flux at the stimulated nodes can be seen by plotting the variance of signal level at all nodes. As Fig. 14 demonstrates, the directly stimulated nodes have a much higher signal variance than unstimulated nodes, although the efflux of particles away from stimulated nodes does cause minor changes in neighbouring nodes.

## Discussion

As demonstrated by the results, HAHF voltage applied between two electrodes in a network of protoplasmic tubes of *P. polycephalum* causes abandonment of the stimulated tube while not affecting the unstimulated tubes. Likewise it was demonstrated that LALF voltage applied between two electrodes in the network maintains the stimulated tube and encourages abandonment of other tubes. As shown in Figs 3 and 4, even the resistance of the stimulated tube increases over time, however the increase is marginal and can be attributed to tube aging and or drying as this occurs in all tubes regardless of stimulation type.

There are an vast number of combinations of frequency and amplitude which could be tested for response to stimulation; this is a task which is to be the subject of future work, as different combinations may produce a diverse range of responses. Stepwise changes in frequency and amplitude will be performed to investigate any other phenomena which could be useful in a future PhyChip; it is acknowledged that it is not possible to test every combination of frequency due to the time taken to test just a single combination. It is probable that there will be an upper and lower limit of frequency, beyond which there will be no more noticeable affect; likewise an upper limit on amplitude, above which the organism will likely die.

The custom Petri-dish is far from ideal, as manufacture is time consuming and satisfactory growth is not always achieved. Multi-electrode arrays (MEA), also called micro-electrode arrays are devices which contain several recording electrodes, often in the space of several millimetres square; they usually have a central well for cell culture and surface adhesion. Typically MEAs are used for recording neuronal electrical activity at multiple sites within a small area; when neurons are excited, ionic currents are generated through the cell membrane which can be detected as a change in voltage between several of these electrodes. It is envisaged that instead of using the large custom Petri-dish, that a custom MEA could be used for *P. polycehpalum* network growth and subsequent measurement and stimulation. The size of the MEA would need to be larger than that of the neuronal MEAs, because one protoplasmic tube could cover the same area as the whole mass of neurons; this may require a custom MEA design with an area of several centimetres square and the possibility of agar as a supporting medium.

The associated response to negative and positive stimuli in the form of voltage application, can be regarded as a form of learning. Observing the stimuli-response correlation, it could be said that voltage stimulation in networks of protoplasmic tubes of *Physarum polycephalum* is equivalent to negative and positive reinforcement learning; connectivity in the network is directly correlated to applied stimulation. Abandonment of tubes is a behavior which is caused by conditioning. Tubes can be abandoned and then regrow to reform connections; this has been observed by the authors; it is possible that the learned state could be changed or reset by changing localised conditions such as light or temperature to initiate regrowth, or even reinforce the learning performed. One drawback to this method is the time taken to regrow tubes, typically a few hours, however this is a living system, much like the plasticity of neurons in the brain, rather than electronic solid state hardware which can be reset in a matter of milliseconds. The biggest limitation of this biological hybrid technology is the time taken for the system to learn; the stimulation time of several hours is currently a limiting factor. Future work will also be aimed at minimising this time.

These responses to negative and positive stimuli are more clearly visible in the modelling experiments. This is perhaps because adaptation of the model networks are not constrained by adhesion of the slime capsule in the *Physarum* plasmodium. There is avoidance of −ve stimuli in the model which slowly reverses after the stimuli are withdrawn. With very strong −ve stimuli the model networks withdraw completely from the site and do not return after withdrawal of the stimuli. This is suggestive of negative reinforcement learning. For +ve stimuli there is an aggregation of the model population towards the stimulated nodes during +ve stimuli. After the stimulus is withdrawn, however, the population withdraws from the stimulated nodes and is distributed around nearby nodes. On repeated application of +ve stimuli we found that the baseline population level of the stimulated node locations gradually increased after each stimulus period. This might correspond to a spatially implemented form of positive reinforcement learning.

It is difficult to directly relate the potential spatial learning effects of the *Physarum* plasmodium to neurally implemented learning. The amorphous nature of the plasmodium and the homogeneous nature of its constituent parts suggest that different learning mechanisms might be at work. The work of Reid^59^ suggests an environmentally mediated spatial learning and the work within this report suggests possible stimulus response effects and spatial aggregation effects that may also play a role. Nevertheless, recent advances in the understanding of electrical synapses as mediators of network connectivity during learning and memory^60^,^61^,^62^,^63^, and extension of these paradigms to non-neural tissues including bone and pancreas^17^,^20^,^21^ suggest that important parallels may exist. Future work will compare the results of *in vitro* learning in cultured neural networks^64^,^65^,^66^ with circuits composed of Physarum.

With more complex equipment, multiple tubes could be stimulated simultaneously, with negative and positive voltage stimulation; this could have the advantage of complex input-output processing. Much like the logic gates or geometric computation which can be performed by protoplasmic tubes, this system could enable the advancement of the first Physarum Chip; a processor whose processing power is derived from real biological components and their inherent learning ability with negative and positive reinforcement. If the network of protoplasmic connectivity could be trained and retrained with negative and positive reinforcement in the form of HAHF and LALF voltage stimulation, then the possibilities for computation are broad; geometric processing could be performed using inputs as weighted training functions, multiple input combination logic gates could be implemented, in a matter of hours, not including the time taken to produce the PhyChip. Protoplasmic tubes are self repairing and can occasionally reconnect or reconfigure themselves after trauma, so the possibility of an adaptive and reconfigurable network or protoplasmic tubes appears feasible from the findings presented here.

The mechanism of action for HAHF and LALF voltage stimulation is unknown. The most likely cause is that the voltage affects the trans-membrane voltage and therefore the voltage gated ion channels which control movement, environment sensing or decision making. It is possible that HAHF and LALF operate in different ways, for example it has been observed by the authors that applying high direct current voltages to a tube causes it to stop being conductive after a short time, possibly due to the tube being burnt with the high level of current. HAHF could simply be burning the stimulated tubes causing the organism to abandon this tube in the network and increase growth elsewhere in the network. It is however unclear why a LALF waveform would act in the opposite way and cause abandonment of non-stimulated tubes.

Our findings share many features with results reported from stimulating dissociated neuronal cell cultures grown on multi-electrode arrays. For example, Jimbo *et al*.^67^ reported how volleys of electrical stimulation can induce excitatory, inhibitory or neutral responses to subsequent electrical stimulation. This ability has subsequently been exploited for simple pattern recognition^68^.

For more complex scenarios, Bull *et al*.^69^ have suggested the use of machine learning techniques to control stimulation to induce required behaviour, using reinforcement learning in particular. The approach was shown able to control both cultured neurons and a non-linear chemical system. Future work with larger Physarum Chips will explore machine learning control of both the type and spacing of stimulation. This work has demonstrated an amorphous system which is capable of learning and adapting to negative and positive stimulation by rearranging network connectivity; this is inherently similar to the machine learning approach developed by Bull *et al*.^69^ using a neuronal culture grown on an MEA dish; stimulating the neuronal culture produced a “pathway-dependant plasticity”. The similarities between this and the results produced in this article are clear; positive and negative stimulation causes changes in network connectivity on a grid of electrodes which is classified as learning.

## Additional Information

**How to cite this article**: Whiting, J. G. H. *et al*. Towards a Physarum learning chip. *Sci. Rep*. **6**, 19948; doi: 10.1038/srep19948 (2016).
